# The correlation between red blood cell distribution width to serum albumin ratio and all-cause mortality in critically ill patients with chronic obstructive pulmonary disease: a retrospective study using the MIMIC-IV database

**DOI:** 10.3389/fmed.2025.1604615

**Published:** 2025-06-24

**Authors:** Qianqian Zhang, Ling Liu, Jing Zhou, Gang Teng, Yuanyuan Wang, Nianzhi Zhang

**Affiliations:** ^1^The First Clinical Medical College of Anhui University of Chinese Medicine, Heifei, China; ^2^Department of Respiratory Medicine, The First Affiliated Hospital of Anhui University of Chinese Medicine, Heifei, China

**Keywords:** red blood cell distribution width, serum albumin, chronic obstructive pulmonary disease, mortality rate, MIMIC-IV database

## Abstract

**Background:**

Chronic obstructive pulmonary disease (COPD) significantly contributes to critical illness and mortality in intensive care units (ICU), yet validated prognostic biomarkers are limited. The red blood cell distribution width to albumin ratio (RAR), which reflects systemic inflammation and nutritional imbalance, has shown predictive value in chronic kidney disease and diabetes. However, its association with mortality in critically ill COPD patients has not been explored. This study investigates the relationship between RAR and all-cause mortality in this high-risk population.

**Methods:**

Utilizing the MIMIC-IV database, 3,779 patients admitted to the ICU for the first time and diagnosed with COPD between 2008 and 2019 were included. Patients were categorized into four groups (Q1-Q4) based on the RAR quartiles within 24 h of admission. Kaplan–Meier curves and Cox proportional hazards models were employed to analyze the correlation between RAR and all-cause mortality at 30, 90, 180, and 365 days. The dose–response relationship was assessed using restricted cubic splines (RCS), and subgroup sensitivity analyses were conducted to evaluate the robustness of the results.

**Results:**

Patients in the highest RAR group (Q4, 7.18–8.38) exhibited higher baseline inflammation and disease severity. The 30-day and 365-day mortality rates were 31.47 and 36.33%, respectively, significantly higher than those in the Q1 group (6.67 and 8.58%). After multivariate adjustment (age, gender, SOFA/APSIII scores, etc.), the 30-day mortality risk in the Q4 group was 2.13 times greater than that in the Q1 group (HR = 2.13, 95%CI: 1.54–2.99), and the 365-day risk was 2.17 times greater (HR = 2.17, 95%CI: 1.61–2.93). RCS indicated a linear positive correlation between RAR and mortality rates (non-linearity *p* > 0.05), and sensitivity analyses suggested that the trend of increased mortality risk with higher RAR was consistent across different subgroups of age, gender, mechanical ventilation status, and comorbidities (interaction *p* > 0.05), indicating the universality of the predictive efficacy.

**Conclusion:**

RAR serves as an independent predictor of all-cause mortality in critically ill patients with COPD. As a low-cost and readily available biomarker, this index has the potential to provide new insights for risk stratification of ICU patients. However, further prospective studies are needed to confirm its clinical translation value.

## Introduction

Chronic obstructive pulmonary disease (COPD) is a prevalent respiratory disorder characterized by its preventable and manageable features, yet it remains a leading cause of morbidity and mortality worldwide. The disease manifests as progressive airflow limitation and persistent respiratory symptoms, with acute exacerbations (AECOPD) triggered by external stimuli such as infections. AECOPD exacerbates airway inflammation, leading to severe respiratory infections, increased sputum production, dyspnea, and irreversible lung function decline, ultimately resulting in life-threatening respiratory failure (1). Epidemiological data reveal that COPD affects approximately 100 million individuals in China (2), with a prevalence of 8.2% among adults aged ≥40 years. Annually, COPD accounts for 1.28 million deaths and 5–10 million disability cases nationally, and it is projected to become the third leading global cause of death by 2030 (3), imposing immense healthcare and economic burdens. Critically ill patients with COPD admitted to intensive care units (ICUs) often present with complex clinical profiles. Studies indicate that COPD patients in ICUs face elevated mortality risks (4); however, validated prognostic biomarkers for this population remain scarce. Identifying and managing risk factors are critical to reducing mortality in this vulnerable cohort. RAR is the ratio of Red Blood Cell Distribution Width (RDW) to Serum Albumin (ALB). RDW is a readily available biomarker that is considered to have predictive value in many diseases and organ dysfunctions (5–7). Serum ALB typically reflects nutritional status and is negatively correlated with clinical adverse outcomes; lower serum ALB levels during ICU hospitalization are associated with higher risks of infection and death (8). RAR combines the characteristics of RDW and ALB and is applied to assess adverse outcomes related to certain diseases. For instance, Hiroshi et al. (9) found that RAR’s ability to evaluate the prognosis of end-stage renal disease is superior to RDW, with higher RAR values correlating with poorer renal outcomes in patients with Chronic Kidney Disease (CKD). Chen et al. (10) discovered that RAR is an independent predictor of Diabetic Kidney Disease (DKD) in patients with Diabetes Mellitus (DM). RAR is a cost-effective and easily accessible biomarker that may have the potential for risk stratification of DKD. Furthermore, a retrospective study on asthma patients found (11) that there is no nonlinear relationship between RAR and asthma risk, with the risk of asthma increasing linearly as RAR values rise. Additionally, related studies suggest that RAR can predict the prognosis of diseases such as Rheumatoid Arthritis (12), Diabetic Retinopathy (13), and Cancer (14). However, no studies have reported on its relationship with Chronic Obstructive Pulmonary Disease, hence this study aims to analyze the connection between RAR and critical COPD and to reveal the predictive value of RAR for mortality in COPD.

## Methods

### Data and sample sources

This study conducted a retrospective analysis using the fourth-generation database of critical care medical information, MIMIC-IV2.2. The database was developed jointly by the Massachusetts Institute of Technology (MIT) Computer Science and Artificial Intelligence Laboratory and the Beth Israel Deaconess Medical Center (BIDMC), incorporating significant enhancements such as data updates and table structure optimization. The dataset encompasses the medical trajectories of over 380,000 patients admitted to the BIDMC intensive care unit in Boston, USA, from 2008 to 2019, comprising more than 450,000 hospitalization records. It comprises multidimensional clinical features including demographic information, laboratory test indicators, medication records, continuous vital signs monitoring data, surgical procedure codes, ICD standard diagnostic information, medication treatment plans, and post-discharge survival follow-up. Following review by the BIDMC Institutional Review Board, the study was deemed to meet the criteria for data use exemption. The research team obtained access permission (ID: 14280276) after completing certification through the National Institutes of Health (NIH) Human Subjects Protection Course and the Collaborative Institutional Training Initiative (CITI) training program. The database utilizes dual de-identification techniques, with all PHI (Protected Health Information) removed, adhering to the Health Insurance Portability and Accountability Act (HIPAA) Safe Harbor standards, thereby exempting the need for informed consent.

### Research design and population

**Figure 1:**
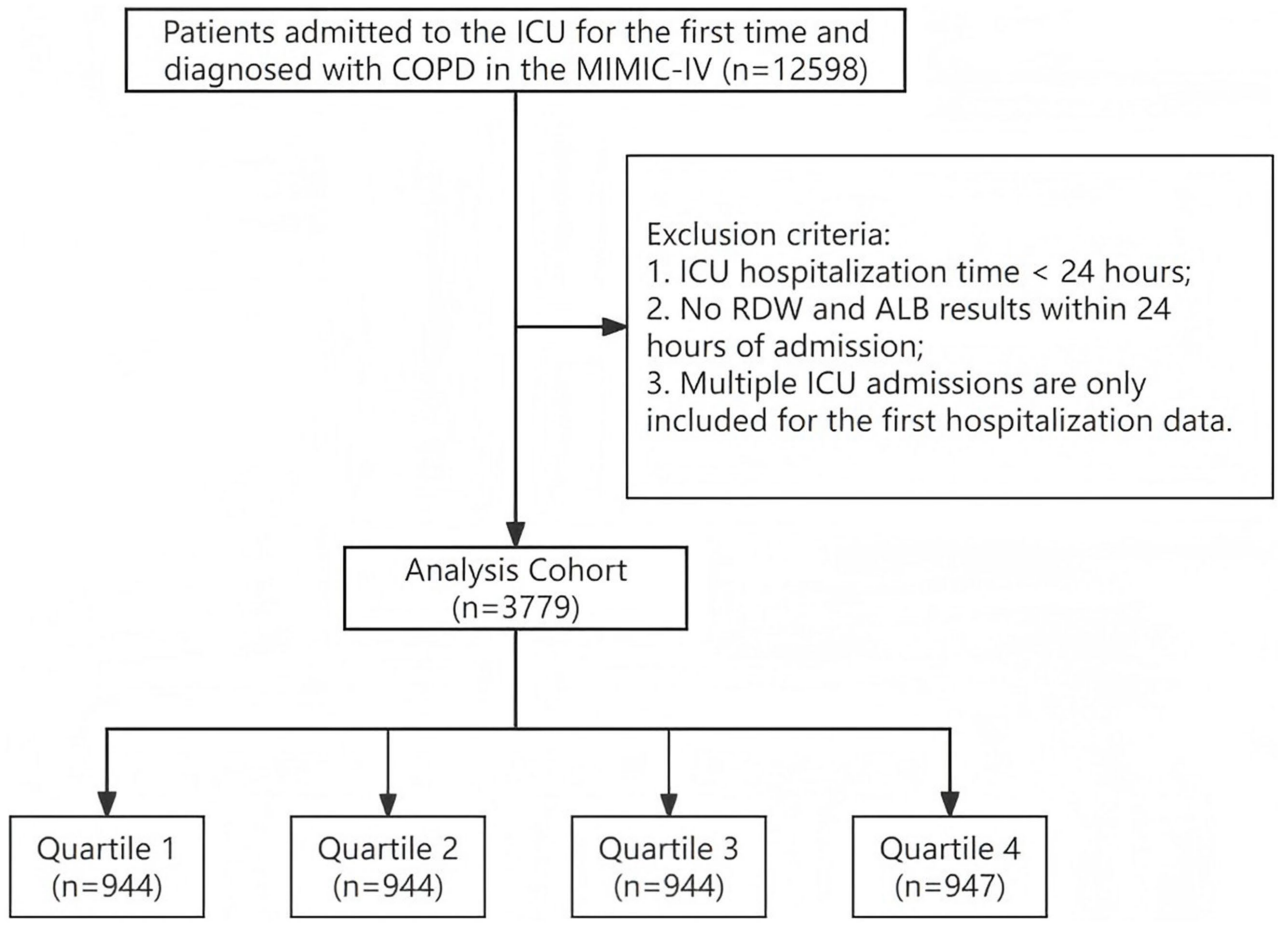
Inclusion of patients in the screening process.

Inclusion Criteria: (1) Age between 18 and 90 years; (2) First admission to the ICU and diagnosed with COPD. Exclusion Criteria: (1) ICU hospitalization time less than 24 h; (2) No RDW and ALB results within 24 h of admission; (3) Patients with multiple ICU admissions are only included for their first admission data. Ultimately, 3,779 patients met the inclusion criteria and were divided into four groups based on the quartiles of RAR (Figure 1).

### Data extraction

Data extraction was performed using Navicat Premium (version 16.1.15) and Structured Query Language (SQL). The study examined various variables categorized as follows: (1) Demographics: age, gender, weight, marital status, ethnicity, language. (2) Past medical history: hypertension (HTN), pneumonia (PNA), cancer (CA), chronic bronchitis (CB), heart failure (HF), coronary heart disease (IHD). (3) Initial vital signs upon ICU admission: heart rate (HR), blood pressure parameters (systolic blood pressure SBP/diastolic blood pressure DBP/mean arterial pressure MBP), respiratory rate (RR), body temperature (T), and oxygen saturation (SpO2). (4) Laboratory indicators: including blood gas analysis (tCO2, iCa, Lac, PaCO2, pH, PaO2), red cell distribution width (RDW), serum albumin (ALB), complete blood cell count (red blood cells, white blood cells, platelets), blood glucose, and electrolytes (Na+, K+, anion gap). (5) Severity of illness scores: Sequential Organ Failure Assessment (SOFA), Acute Physiology Score III (APS III). (6) Outcome indicators: in-hospital mortality, ICU mortality, mortality rates at 30/90/180/365 days. (7) Mechanical ventilation: whether mechanical ventilation was performed, duration of mechanical ventilation. The RAR index, specifically defined, was calculated using the ratio of red cell distribution width to serum albumin (RDW/ALB). During the data preprocessing phase, variables with a missing rate exceeding 20% were excluded, and the remaining missing values were handled through multiple imputation using the random forest algorithm. To ensure data comparability, all laboratory test data were based on the first measurement results within 24 h of admission.

## Results

The primary outcomes of this study were all-cause mortality at 30 and 365 days, while the secondary outcomes included all-cause mortality at 90 and 180 days.

### RAR

The RAR calculation formula is RDW(%)/ALB(g/dl), with RDW and ALB data being directly sourced from MIMIC-IV.

### Statistical analysis

This study included 3,779 critically ill patients with COPD from the MIMIC-IV database and categorized them into four groups (Q1-Q4) based on the quartiles of the Red Cell Distribution Width/Serum Albumin Ratio (RAR) at admission. After conducting the Shapiro–Wilk normality test for continuous variables, non-normal data were represented by the median (interquartile range, IQR), and the Kruskal-Wallis test or Mann–Whitney U test was used for comparisons between groups; categorical variables were expressed as frequency (percentage), and differences between groups were assessed using the chi-square test or Fisher’s exact test. Survival analysis employed the Kaplan–Meier curve and Log-rank test to compare mortality differences among different RAR groups, and a multivariate Cox proportional hazards model was used to adjust for age, gender, SOFA score, APSIII score, vital signs (heart rate, mean arterial pressure), and comorbidities (hypertension, pneumonia, etc.) stepwise. Results were reported as hazard ratios (HR) and 95% confidence intervals (CI), and the Cox model was validated for the proportional hazards assumption using Schoenfeld residuals (*p* > 0.05), ensuring model applicability. Restricted cubic spline (3 knots) analysis was used to analyze the dose–response relationship between RAR and mortality risk. Sensitivity analysis was performed through subgroup interaction tests: stratified by age (≤65 years vs. >65 years), gender, mechanical ventilation status, and comorbidities (malignancy, coronary heart disease), to assess the interaction between RAR and mortality risk (interaction term *p* < 0.05 was considered significant). All analyses were performed using DecisionLinnc 1.0 software (15), with the significance level set at two-sided *p* < 0.05, and all results were verified multiple times to ensure the accuracy and robustness of the analysis.

## Results

### Baseline characteristics of the study subjects

This study analyzed data from 12,598 patients in the MIMIC-IV database, of which 3,779 COPD patients met the inclusion criteria. The average age of the participants was 71.06 years (range: 60.94 to 81.18 years), with 53.69% being male. The data was binned according to the RAR value at admission, divided into quartiles: Q1 (3.68 ≤ RAR < 4.78, *n* = 944), Q2 (4.78 ≤ RAR < 5.32, *n* = 944), Q3 (5.32 ≤ RAR < 7.18, *n* = 944), Q4 (7.18 ≤ RAR < 8.38, *n* = 997). Table 1 shows the baseline characteristics of these groups. The participants in the highest RAR value group (Q4) had longer mechanical ventilation times, and higher prevalence rates of pneumonia (42.98%) and malignant tumors (21.12%). Compared to the other groups, their total calcium, free calcium, PCO2, PH, po2, hemoglobin, RBC, hematocrit, hemoglobin, and serum sodium levels were lower, while lactate, temperature, absolute lymphocyte count, platelet, absolute neutrophil count, and anion_gap levels were higher. In terms of disease severity scores at ICU admission, the Q4 group consistently scored higher in SOFA and APSIII and had the longest ICU stay. Compared to the other groups, the Q4 group had significantly higher mortality rates at all time points: 30-day mortality (31.47% vs. 17.80% vs. 11.23% vs. 6.67%, *p* < 0.001), 90-day mortality (34.74% vs. 19.49% vs. 12.39% vs. 7.20%, *p* < 0.001), 180-day mortality (35.69% vs. 20.02% vs. 12.61% vs. 7.84%, *p* < 0.001), and 365-day mortality (36.33% vs. 20.66% vs. 13.24% vs. 8.58%, *p* < 0.001). ICU mortality rates also gradually increased over time.

**Table 1:** Baseline characteristics of participants.

Variable	Overall	Q1 (3.68 ≤ RAR < 4.78; *n* = 944)	Q2 (4.78 ≤ RAR < 5.32; *n* = 944)	Q3(5.32 ≤ RAR < 7.18; *n* = 944)	Q4(7.18 ≤ RAR < 8.38; *n* = 997)	*p*-value
	*N* = 3,779	*N* = 944	*N* = 944	*N* = 944	*N* = 947	
Age (years)	71.06 ± 10.12	69.99 ± 10.29	71.21 ± 9.99	71.92 ± 9.82	71.13 ± 10.30	<0.001
HospDays (days)	13.18 ± 12.95	8.85 ± 7.31	11.66 ± 11.57	14.48 ± 13.78	17.72 ± 15.83	<0.001
ICUDays (days)	4.92 ± 6.46	3.70 ± 4.21	4.49 ± 5.20	5.25 ± 7.40	6.22 ± 8.01	<0.001
VentHrs (hours)	69.79 ± 96.84	50.29 ± 63.61	63.97 ± 81.64	75.78 ± 108.59	89.06 ± 119.16	<0.001
SOFA(score)	5.07 ± 3.50	3.46 ± 2.64	4.64 ± 3.03	5.48 ± 3.51	6.68 ± 3.89	<0.001
APSIII (score)	45.62 ± 19.64	35.83 ± 13.59	41.61 ± 15.50	47.85 ± 19.32	57.13 ± 22.27	<0.001
TCO2 (mmol/L)	26.80 ± 6.41	27.34 ± 6.09	26.98 ± 5.99	27.15 ± 6.75	25.75 ± 6.64	<0.001
iCa (mmol/L)	1.13 ± 0.18	1.14 ± 0.11	1.14 ± 0.11	1.14 ± 0.30	1.12 ± 0.13	<0.001
Lac (mmol/L)	2.02 ± 1.63	1.80 ± 1.04	1.87 ± 1.30	1.97 ± 1.53	2.42 ± 2.29	<0.001
PaCO2 (mmHg)	48.29 ± 14.01	48.25 ± 13.73	47.97 ± 13.13	49.45 ± 14.83	47.48 ± 14.23	0.027
pH	7.35 ± 0.09	7.36 ± 0.08	7.35 ± 0.09	7.34 ± 0.09	7.33 ± 0.10	<0.001
PaO2 (mmHg)	118.69 ± 105.53	133.80 ± 114.18	127.22 ± 109.73	114.43 ± 103.80	99.35 ± 89.67	<0.001
Temp (°C)	36.87 ± 0.69	36.76 ± 0.70	36.88 ± 0.66	36.93 ± 0.66	36.93 ± 0.72	<0.001
ALC (×10^3^/μl)	1.49 ± 6.73	1.51 ± 1.06	1.69 ± 8.05	1.25 ± 1.11	1.52 ± 10.67	<0.001
Hct (%)	32.42 ± 7.01	36.27 ± 6.44	32.94 ± 6.44	31.10 ± 6.61	29.37 ± 6.65	<0.001
Hgb (g/dL)	10.32 ± 2.30	11.80 ± 2.11	10.53 ± 2.07	9.78 ± 2.09	9.18 ± 2.06	<0.001
Plt (×10^3^/μl)	206.49 ± 101.35	207.66 ± 81.65	202.29 ± 88.47	200.99 ± 96.26	215.01 ± 131.21	0.087
RDW (%)	15.54 ± 2.57	13.60 ± 1.06	14.69 ± 1.40	15.93 ± 1.89	17.92 ± 3.09	<0.001
RBC (×10^6^/μl)	3.51 ± 0.80	3.89 ± 0.73	3.54 ± 0.72	3.38 ± 0.78	3.23 ± 0.82	<0.001
WBC (×10^3^/μl)	12.77 ± 9.57	11.68 ± 7.95	12.01 ± 9.56	12.63 ± 7.66	14.75 ± 12.14	<0.001
ANC (×10^3^/μl)	10.20 ± 6.88	9.16 ± 5.20	9.43 ± 5.25	10.11 ± 7.13	12.08 ± 8.88	<0.001
Alb (g/dL)	3.12 ± 0.57	3.69 ± 0.34	3.28 ± 0.31	2.99 ± 0.36	2.52 ± 0.47	<0.001
AG (mmol/L)	14.01 ± 4.48	13.94 ± 4.17	13.81 ± 4.29	13.82 ± 4.35	14.48 ± 5.05	0.080
K (mmol/L)	4.37 ± 0.79	4.30 ± 0.66	4.36 ± 0.80	4.41 ± 0.83	4.40 ± 0.84	0.052
Na (mmol/L)	137.90 ± 5.35	138.00 ± 4.86	137.97 ± 5.01	138.06 ± 5.62	137.57 ± 5.84	0.022
Gender; *n* (%)						0.053
Female	1;750.00 (46.31%)	413.00 (43.75%)	421.00 (44.60%)	466.00 (49.36%)	450.00 (47.52%)	
Male	2;029.00 (53.69%)	531.00 (56.25%)	523.00 (55.40%)	478.00 (50.64%)	497.00 (52.48%)	
HTN; *n* (%)						<0.001
No	2;437.00 (64.49%)	492.00 (52.12%)	610.00 (64.62%)	643.00 (68.11%)	692.00 (73.07%)	
Yes	1;342.00 (35.51%)	452.00 (47.88%)	334.00 (35.38%)	301.00 (31.89%)	255.00 (26.93%)	
PNA; *n* (%)						<0.001
No	2;518.00 (66.63%)	746.00 (79.03%)	637.00 (67.48%)	595.00 (63.03%)	540.00 (57.02%)	
Yes	1;261.00 (33.37%)	198.00 (20.97%)	307.00 (32.52%)	349.00 (36.97%)	407.00 (42.98%)	
CA; *n* (%)						0.278
No	3;055.00 (80.84%)	774.00 (81.99%)	773.00 (81.89%)	761.00 (80.61%)	747.00 (78.88%)	
Yes	724.00 (19.16%)	170.00 (18.01%)	171.00 (18.11%)	183.00 (19.39%)	200.00 (21.12%)	
HF; *n* (%)						<0.001
No	2;063.00 (54.59%)	639.00 (67.69%)	518.00 (54.87%)	449.00 (47.56%)	457.00 (48.26%)	
Yes	1;716.00 (45.41%)	305.00 (32.31%)	426.00 (45.13%)	495.00 (52.44%)	490.00 (51.74%)	
IHD; *n* (%)						0.003
No	1;985.00 (52.53%)	544.00 (57.63%)	470.00 (49.79%)	484.00 (51.27%)	487.00 (51.43%)	
Yes	1;794.00 (47.47%)	400.00 (42.37%)	474.00 (50.21%)	460.00 (48.73%)	460.00 (48.57%)	
In-hospital mortality; *n* (%)						<0.001
No	3;587.00 (94.92%)	922.00 (97.67%)	903.00 (95.66%)	895.00 (94.81%)	867.00 (91.55%)	
Yes	192.00 (5.08%)	22.00 (2.33%)	41.00 (4.34%)	49.00 (5.19%)	80.00 (8.45%)	
ICU mortality; *n* (%)						<0.001
No	3;182.00 (84.20%)	880.00 (93.22%)	848.00 (89.83%)	786.00 (83.26%)	668.00 (70.54%)	
Yes	597.00 (15.80%)	64.00 (6.78%)	96.00 (10.17%)	158.00 (16.74%)	279.00 (29.46%)	
30-day ICU mortality; *n* (%)						<0.001
No	3;144.00 (83.20%)	881.00 (93.33%)	838.00 (88.77%)	776.00 (82.20%)	649.00 (68.53%)	
Yes	635.00 (16.80%)	63.00 (6.67%)	106.00 (11.23%)	168.00 (17.80%)	298.00 (31.47%)	
90-day ICU mortality; *n* (%)						<0.001
No	3;081.00 (81.53%)	876.00 (92.80%)	827.00 (87.61%)	760.00 (80.51%)	618.00 (65.26%)	
Yes	698.00 (18.47%)	68.00 (7.20%)	117.00 (12.39%)	184.00 (19.49%)	329.00 (34.74%)	
180-day ICU mortality; *n* (%)						<0.001
No	3;059.00 (80.95%)	870.00 (92.16%)	825.00 (87.39%)	755.00 (79.98%)	609.00 (64.31%)	
Yes	720.00 (19.05%)	74.00 (7.84%)	119.00 (12.61%)	189.00 (20.02%)	338.00 (35.69%)	
365-day ICU mortality; *n* (%)						<0.001
No	3;034.00 (80.29%)	863.00 (91.42%)	819.00 (86.76%)	749.00 (79.34%)	603.00 (63.67%)	
Yes	745.00 (19.71%)	81.00 (8.58%)	125.00 (13.24%)	195.00 (20.66%)	344.00 (36.33%)	

SOFA, Sequential Organ Failure Assessment; APSIII, Acute Physiology Score III; TCO2, Total carbon dioxide; iCa, Ionized calcium; Lac, Lactate; PaCO2, Partial pressure of carbon dioxide; PaO2, Partial pressure of oxygen; Temp, Temperature; ALC, Absolute lymphocyte count; Hct, Hematocrit; Hgb, Hemoglobin; Plt, Platelet count; RDW, Red blood cell distribution width; RBC, Red blood cell count; WBC, White blood cell count; ANC, Absolute neutrophil count; Alb, Albumin; AG, Anion gap; K, Potassium; Na, Sodium; HTN, Hypertension; PNA, Pneumonia; CA, Malignancy; HF, Heart failure; IHD, Ischemic heart disease.

### Clinical outcomes

**Figure 2:**
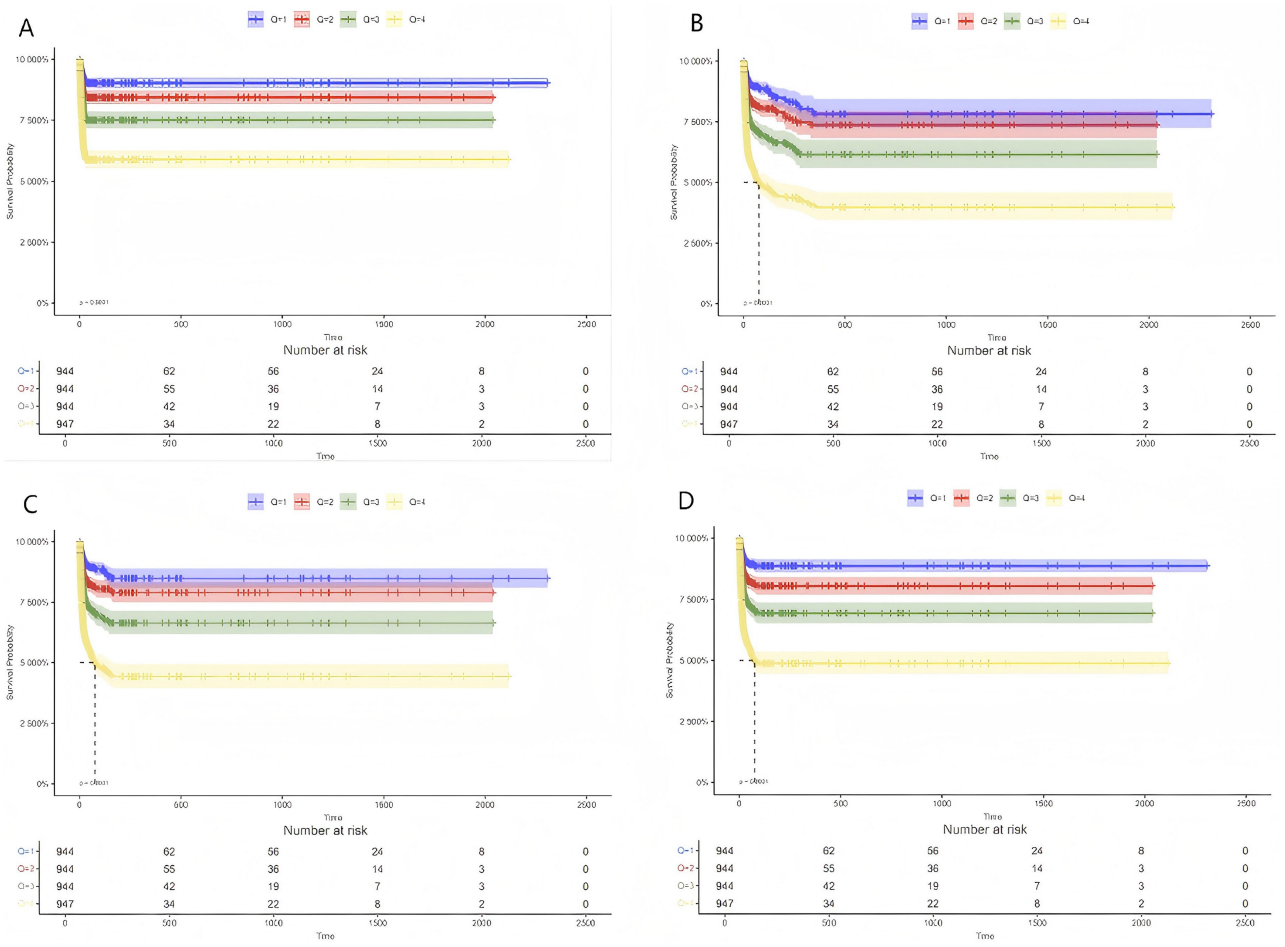
Kaplan–Meier survival analysis curve for all-cause mortality rate. Kaplan–Meier curves for all-cause mortality at 30 days **(A)**, 90 days **(B)**, 180 days **(C)**, and 365 days **(D)** stratified by RAR value.

The Kaplan–Meier curve (Figure 2) illustrates the disparities in mortality rates at 30, 90, 180, and 365 days across the quartiles of RAR. In comparison to the lower RAR index groups, patients in the highest RAR index group (Q4) demonstrated significantly decreased survival rates at 30, 90, 180, and 365 days (log-rank *p* < 0.01). The remaining three groups (Q1, Q2, and Q3) also displayed variations in survival rates at each time point (30, 90, 180, and 365 days).

To investigate the independent impact of RAR on mortality, two Cox regression models were employed (Tables 2 and 3). After controlling for age, gender, SOFA score, and APSIII score (Model 2), the hazard ratios (HR) and 95% confidence intervals (CIs) for the RAR index categories (3.68–4.78, 4.78–5.32, 5.32–7.18, 7.18–8.38) were as follows: for 30-day all-cause mortality, the HR was 1.00 (reference), and 1.18 (0.84–1.67), 1.47 (1.06–2.05), and 2.37 (1.73–3.28), respectively. After adjusting for age, gender, body temperature, PaCO2, PaO2, Lac, pH, SOFA score, APSIII score, hypertension, pneumonia, heart failure, and coronary heart disease (Model 3), the HR and 95% CIs for the RAR index categories (3.68–4.78, 4.78–5.32, 5.32–7.18, 7.18–8.38) were as follows: for 30-day all-cause mortality, the HR was 1.00 (reference), and 1.13 (0.79–1.61), 1.39 (1.00–1.96), and 2.13 (1.54–2.99), respectively. For 365-day all-cause mortality, the HRs in Model 2 were 1.00 (reference), 1.13 (0.83–1.55), 1.45 (1.08–1.96), and 2.46 (1.85–3.31), respectively. The HRs in Model 3 were 1.00 (reference), 1.06 (0.77–1.46), 1.35 (1.00–1.84), and 2.17 (1.61–2.93), respectively. These results indicate that patients with RAR ≥ 7.18 face a higher risk of 30-day and 365-day all-cause mortality compared to those with RAR < 7.18. A similar pattern was observed for 90-day and 180-day all-cause mortality; details are available in Supplementary Tables 1, 2.

**Table 2:** COX regression model (30-day all-cause mortality rate).

Variables	Model 1		Model 2		Model 3	
	HR (95%CI)	*p* value	HR (95%CI)	*p* value	HR (95%CI)	*p* value
RAR quantile						
Q1	1.00 (Reference)		1.00 (Reference)		1.00 (Reference)	
Q2	1.77 (1.28–2.46)	<0.001	1.18 (0.84–1.67)	0.338	1.13 (0.79–1.61)	0.504
Q3	3.03 (2.24–4.13)	<0.001	1.47 (1.06–2.05)	0.022	1.39 (1.00–1.96)	0.054
Q4	6.42 (4.84–8.65)	<0.001	2.37 (1.73–3.28)	<0.001	2.13 (1.54–2.99)	<0.001

**Table 3:** COX regression model (365-day all-cause mortality rate).

Variables	Model 1		Model 2		Model 3	
	HR (95%CI)	*p* value	HR (95%CI)	*p* value	HR (95%CI)	*p* value
RAR quantile						
Q1	1.00 (Reference)		1.00 (Reference)		1.00 (Reference)	
Q2	1.63 (1.21–2.19)	0.001	1.13 (0.83–1.55)	0.437	1.06 (0.77–1.46)	0.709
Q3	2.77 (2.11–3.68)	<0.001	1.45 (1.08–1.96)	0.014	1.35 (1.00–1.84)	0.056
Q4	6.08 (4.69–7.95)	<0.001	2.46 (1.85–3.31)	<0.001	2.17 (1.61–2.93)	<0.001

### Detection of nonlinear relationships

**Figure 3:**
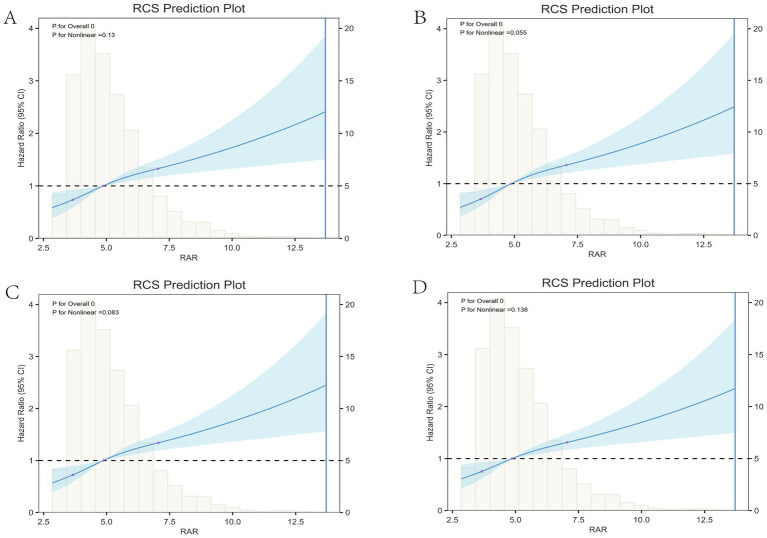
RCS of RAR with all-cause mortality. RCS of RAR with 30-day **(A)**, 90-day **(B)**, 180-day **(C)** and 365-day **(D)** all-cause mortality.

The analysis using Restricted Cubic Spline (RCS) curves (Figure 3) indicated no nonlinear relationship between RAR values and all-cause mortality at various time points: 30 days (*p* for nonlinearity = 0.13), 90 days (*p* for nonlinearity = 0.055), 180 days (*p* for nonlinearity = 0.083), and 365 days (*p* for nonlinearity = 0.138). These results collectively demonstrate a linear positive association between RAR (as a continuous variable) and mortality risk (*p* for nonlinearity > 0.05 for all intervals).

### Sensitivity analysis

**Figure 4:**
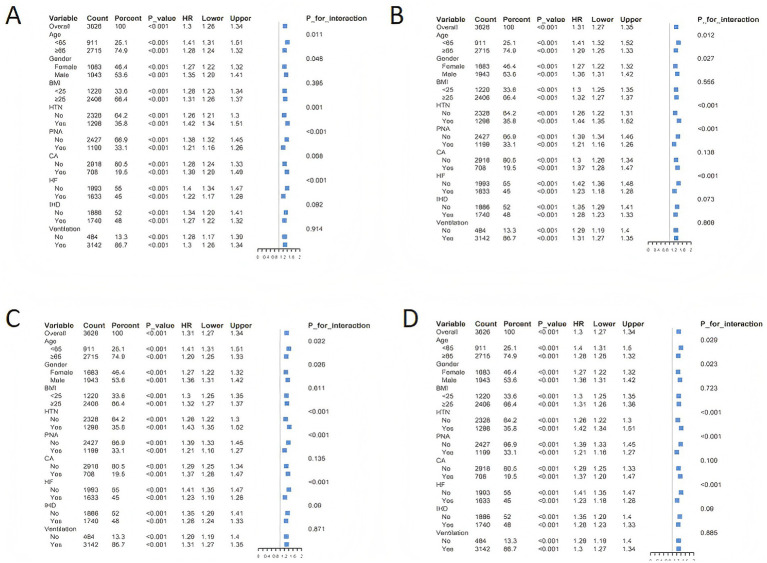
Forest plot of stratified analysis of all-cause mortality rates by RAR value and 30 days **(A)**, 90 days **(B)**, 180 days **(C)**, and 365 days **(D)**. BMI, Body Mass Index; HTN, Hypertension; PNA, Pneumonia; CA, Cancer; HF, Heart Failure; IHD, Ischemic Heart Disease.

The results of the stratified analysis of RAR values with all-cause mortality at 30 days (A), 90 days (B), 180 days (C), and 365 days (D) have been summarized. From Figure 4, it is observed that mechanical ventilation, history of malignant tumor, and history of coronary heart disease do not interact with RAR. This suggests that irrespective of mechanical ventilation or comorbidities such as malignant tumor or coronary heart disease, the risk of death in critically ill COPD patients increases with higher RAR values. Age, gender, history of hypertension, history of pneumonia, and history of heart failure do interact with RAR, but in all cases, the risk of death in critically ill COPD increases with the increase of RAR. In summary, all subgroups in this study align with the general population trend, indicating that an increase in RAR is a risk factor for death in critically ill COPD patients.

## Discussion

This study has, for the first time, revealed a significant association between RAR and all-cause mortality in critically ill patients with COPD. Through the analysis of 3,779 patients from the MIMIC-IV database, we found that elevated RAR levels were independently and positively correlated with the risk of all-cause mortality at 30, 90, 180, and 365 days, and this association was dose-dependent. Even after adjusting for age, severity of illness scores (SOFA, APSIII), and various confounding factors, the highest RAR quartile group (Q4) still showed a 2.13-fold increase in 30-day mortality risk and a 2.17-fold increase in 365-day mortality risk compared to the lowest quartile group (Q1). These findings suggest that RAR may serve as a novel biomarker for prognosis in critically ill COPD patients, with significant clinical translational value.

### The pathophysiological significance and mechanism of RAR

RDW is an indicator that reflects the heterogeneity of red blood cell size, and its diagnostic and prognostic value in various diseases has garnered widespread attention in recent years. Studies have found (16, 17) that RDW can effectively assess the deterioration of lung function in COPD patients and serves as an independent predictor of prognosis. Elevated RDW is closely associated with chronic inflammation, oxidative stress, and abnormal erythropoiesis (18, 19). COPD patients commonly exhibit a chronic inflammatory state, characterized by persistent activation of airway and systemic inflammatory responses. This chronic inflammation can lead to increased RDW through multiple mechanisms. First, inflammatory cytokines such as IL-6 and TNF-*α* can inhibit the erythropoietin (EPO) signaling pathway, thereby suppressing bone marrow erythropoiesis (20). EPO is a key regulator of erythropoiesis, and its reduced bioactivity can impair red blood cell production, leading to the release of immature red blood cells and ultimately manifesting as an elevated peripheral blood RDW. Secondly, AECOPD patients often experience hypoxemia, with severe cases complicated by respiratory failure. Hypoxia can upregulate the expression of HIF-1α in red blood cells, activating EPO gene transcription and stimulating erythropoiesis (21). Under severe hypoxia, immature red blood cells may be mobilized and released, resulting in an increased peripheral blood RDW. Lastly, most COPD patients suffer from malnutrition, with insufficient intake and impaired absorption of iron, folate, and vitamin B12, leading to reduced hemoglobin synthesis substrates and increased red blood cell heterogeneity, ultimately elevating RDW (22). ALB, a nutrient synthesized by the liver, is involved in substance transport and promotes the immune function of immunoglobulins. Consequently, alterations in ALB levels not only reflect the body’s nutritional status but also its inflammatory and immune conditions. Lowered ALB levels suggest malnutrition, inflammatory activation, and compromised liver function (23). Research has indicated that patients with AECOPD often experience stress and hypercatabolism, with increased oxygen consumption by respiratory muscles, endocrine dysfunction, and tissue damage from viruses or bacteria, all of which impact ALB synthesis (24). Moreover, AECOPD primarily affects middle-aged and elderly individuals, whose appetite can be influenced by systemic function, medication use, and psychological state, further diminishing liver nutrient supply and leading to a decrease in ALB levels, thereby impairing immune function (25). When inflammatory responses worsen, RDW increases while ALB decreases, resulting in an elevated RDW/ALB ratio(RAR), which suggests aggravated systemic inflammation, impaired immune function, and poor nutritional status in COPD patients. Chronic hypoxia in COPD patients often leads to abnormal erythropoiesis, and elevated RDW may reflect bone marrow stress. Meanwhile, hypoalbuminemia is associated with the inflammatory burden during COPD exacerbations. In COPD patients, chronic systemic inflammation and recurrent acute exacerbations exacerbate oxidative stress, further damaging pulmonary vascular endothelium and promoting increased red blood cell heterogeneity. Inflammatory cytokines (e.g., IL-6, TNF-*α*) also suppress hepatic albumin synthesis, reducing ALB levels (26). An elevated RAR may reflect the intensification of this vicious cycle, indicating that patients are in a pathological state of “high inflammation-high oxidative stress-malnutrition.” Furthermore, hypoalbuminemia may weaken immune function and increase infection risk, while high RDW may exacerbate tissue hypoxia, collectively contributing to organ dysfunction and elevated mortality risk.

### Comparison with existing research and innovation

Previous studies have confirmed the independent prognostic value of RDW and ALB in COPD outcomes (27, 28), but the combined application of RAR has not yet been explored. This study is the first to introduce their ratio into risk stratification for critically ill COPD patients, demonstrating its superior predictive performance compared to individual indicators. For instance, Chen et al.’s research on diabetic nephropathy showed that RAR’s predictive capability outperformed RDW, which aligns with our findings. Additionally, the linear association characteristic of RAR (no nonlinear trend observed in RCS analysis) simplifies threshold setting for clinical application, while the absence of interaction effects in sensitivity analyses (mechanical ventilation, malignant tumors) suggests robust predictive stability. Notably, although SOFA and APSIII scores already incorporate partial organ function information, RAR still provides independent incremental prognostic value, likely because the systemic inflammation-nutrition imbalance state it reflects is not fully captured by traditional scoring systems.

### Clinical significance and potential applications

RAR, being a cost-effective and easily accessible indicator, can aid ICU doctors in rapidly identifying high-risk COPD patients. For patients with RAR ≥ 7.18, it is recommended to intensify monitoring and initiate early intervention, such as optimizing nutritional support (e.g., albumin supplementation), controlling inflammatory responses (e.g., rational use of glucocorticoids), and correcting anemia. Furthermore, dynamic monitoring of RAR can assist in evaluating treatment responses. For example, a decrease in RAR with treatment may suggest inflammation relief and an improvement in nutritional status, whereas an increase should prompt concerns about disease progression.

## Research limitations and future directions

This study has several limitations: First, despite rigorous adjustments for clinically relevant confounding factors using multivariate Cox models, residual confounding may persist due to unmeasured factors (e.g., pack-years of smoking, inhaled corticosteroid regimens, GOLD stages). Given that smoking history is a significant driver of systemic inflammation and COPD progression (29), and inhaled corticosteroids may affect RDW through hematopoietic modulation (30), these omitted factors could theoretically introduce estimation bias. However, the limitations of the MIMIC-IV database concerning outpatient treatment records make such adjustments challenging. Future prospective studies that include detailed pulmonary function tests and treatment histories are necessary for validation. Second, the MIMIC-IV data originates from a single medical center, which may introduce selection bias. Thirdly, while this study concentrated on baseline RAR measurements, emerging evidence indicates that dynamic biomarker monitoring can enhance prognostic accuracy (31). A clinical trial involving sepsis patients showed that continuous RDW/ALB ratio monitoring significantly improved mortality prediction compared to isolated measurements (32). Future research should assess the dynamic alterations in RAR throughout ICU hospitalization, especially in relation to ventilator weaning protocols and nutritional interventions. Additionally, the absence of an external validation cohort presents a limitation. Future multicenter prospective studies should include pulmonary function tests, imaging, and biomarkers (such as CRP, IL-6) to further validate the predictive value of RAR and to explore its correlation with COPD phenotypes (for example, the frequent exacerbation phenotype). Mechanistic research could concentrate on the molecular pathways that connect RAR to pulmonary vascular remodeling and immunometabolic regulation.

## Conclusion

RAR serves as an independent predictor of all-cause mortality in critically ill patients with COPD, and its elevation is closely associated with inflammatory activation, nutritional imbalance, and increased oxidative stress. This indicator offers new insights for risk stratification and precise management of COPD, and warrants further validation and application in clinical practice.

## Data Availability

The original contributions presented in the study are included in the article/Supplementary material, further inquiries can be directed to the corresponding author.
